# Xerostomia Induced by Psychiatric Medications: Prevalence, Impact, and Management

**DOI:** 10.1192/j.eurpsy.2025.2088

**Published:** 2025-08-26

**Authors:** R. A. Maldonado-Puebla, M. Murugappan, B. Carr

**Affiliations:** 1 College of Osteopathic Medicine, Nova Southeastern University, Clearwater; 2 Psychiatry, University of Florida, Gainesville, United States

## Abstract

**Introduction:**

Xerostomia, or dry mouth caused by reduced salivary flow, is a frequently reported adverse effect of various psychiatric medications, particularly tricyclic antidepressants (TCAs), antipsychotics, SSRIs, SNRIs, and anticholinergics. This condition can lead to severe oral health problems causing many patients to discontinue their medication. Anticholinergics and psychotropic medications cause xerostomia by blocking acetylcholine from binding to muscarinic receptors in the salivary glands. Determining rates of xerostomia among psychotropic medications could be useful for those who are at higher risk of xerostomia.

**Objectives:**

To investigate the prevalence and clinical impact of xerostomia caused by psychiatric medications and to identify effective management strategies.

**Methods:**

A narrative literature review of clinical trials, observational studies, and case reports was performed to gather data on the prevalence, severity, and management of xerostomia in patients on psychiatric medications. The review included TCAs (amitriptyline, nortriptyline), antipsychotics (clozapine, olanzapine, chlorpromazine), SSRIs (paroxetine, fluoxetine, sertraline, citalopram), SNRIs (venlafaxine, duloxetine), and anticholinergics (benztropine, trihexyphenidyl). Patient-reported outcomes and interventions were analyzed.

**Results:**

Table 1: Prevalence of Xerostomia by Medication

**Table:** 

Medication	Prevalence (%)	Severity (Mild/Moderate/Severe)	Management Strategies
Amitriptyline	30-50	Moderate to Severe	Saliva Substitutes, Pilocarpine
Clozapine	10-30	Mild to Moderate	Dose Reduction, Hydration
Paroxetine	20-40	Mild to Moderate	Sugar-free Chewing Gum, Hydration
Anticholinergics*	20-65	Moderate to Severe	Dose Adjustment, Saliva Substitutes

*Anticholinergics include benztropine and trihexyphenidyl, commonly used to manage extrapyramidal symptoms.

The prevalence of xerostomia was highest with amitriptyline (30-50%), followed by paroxetine (20-40%) and clozapine (10-30%). Anticholinergics contributed to xerostomia in 20-65% of cases. Amitriptyline and anticholinergics often caused moderate to severe cases of xerostomia. Management strategies included the use of saliva substitutes and pilocarpine for TCAs and anticholinergics, dose reduction and increased hydration for antipsychotics, and sugar-free chewing gum for SSRIs. Other medications not listed that are notable for significantly inducing xerostomia among their medication class include citalopram for SSRIs, venlafaxine for SNRIs, and chlorpromazine for antipsychotics.

**Conclusions:**

TCAs and anticholinergics pose the highest risk for the side effect of xerostomia among psychiatric medications. Effective management requires a multifaceted approach, including pharmacologic and non-pharmacologic interventions. Future research should aim to explore alternative medications with lower xerostomia risk.

**Disclosure of Interest:**

None Declared

